# Establishment of *Babesia vulpes* n. sp. (Apicomplexa: Babesiidae), a piroplasmid species pathogenic for domestic dogs

**DOI:** 10.1186/s13071-019-3385-z

**Published:** 2019-03-26

**Authors:** Gad Baneth, Luís Cardoso, Paula Brilhante-Simões, Leonhard Schnittger

**Affiliations:** 1 Koret School of Veterinary Medicine, Hebrew University, P.O. Box 12, 76100 Rehovot, Israel; 2 Department of Veterinary Sciences, and Animal and Veterinary Research Centre (CECAV), University of Trás-os-Montes e Alto Douro (UTAD), Vila Real, Portugal; 3 Inno – Serviços Especializados em Veterinária, Braga, Portugal; 4 Instituto de Patobiología Veterinaria, (CICVyA), Instituto Nacional de Tecnología Agropecuaria (INTA), 1686 Hurlingham, Argentina; 5 Consejo Nacional de Investigaciones Científicas y Técnicas (CONICET), Buenos Aires, Argentina

**Keywords:** *Babesia vulpes* n. sp., *Babesia microti*, *Babesia* cf. *microti*, *Babesia microti*-like piroplasm, *Theileria annae*, “*Babesia* (*Theileria*) *annae”*, *Babesia annae*, “*Babesia* Spanish dog isolate”, Red fox, Dog

## Abstract

**Background:**

Canine babesiosis is a severe disease caused by several *Babesia* spp. A number of names have been proposed for the canine-infecting piroplasmid pathogen initially named *Theileria annae* Zahler, Rinder, Schein & Gothe, 2000. It was shown to be a member of the *Babesia* (*sensu lato*) group infecting carnivores and is also closely related to the *Babesia microti* group. Subsequently, the same parasite species was reclassified as a member of the genus *Babesia* and the name *Babesia vulpes* Baneth, Florin-Christensen, Cardoso & Schnittger, 2015 was proposed for it. However, both names do not meet the requirements of the *International Code of Zoological Nomenclature* (no accompanying descriptions, no deposition of type-specimens) and cannot be recognized as available names from the nomenclatural point of view. The purpose of this study was to further characterize this parasite in order to confirm its validity, to provide its description and to introduce zoological nomenclature for it with the name *Babesia vulpes* n. sp.

**Results:**

Morphological description of the parasite in canine erythrocytes demonstrated that it takes the shape of small (1.33 × 0.98 µm), round to oval forms reminiscent of the pyriform and ring shapes of other small canine *Babesia* spp., such as *Babesia gibsoni* Patton, 1910 and *Babesia conradae* Kjemtrup, Wainwright, Miller, Penzhorn & Carreno, 2006. However, these parasite forms were overall smaller than those measured for the latter two species and no tetrad (Maltese cross) form was reported. Furthermore, phylogenetic analysis using the cytochrome *c* oxidase subunit 1 (COX1) amino acid sequences substantiates the species identity of this parasite as previously demonstrated based on phylogenetic analysis of the *18S* rRNA and *β-tubulin* genes. The holotype of the parasite species was designated and deposited in an accessible public collection.

**Conclusions:**

This study ratifies the name *Babesia vulpes* n. sp. proposed for the parasite previously referred to as *Theileria annae* Zahler, Rinder, Schein & Gothe, 2000, *Babesia annae* (Zahler, Rinder, Schein & Gothe, 2000) or *Babesia vulpes* Baneth, Florin-Christensen, Cardoso & Schnittger, 2015, or mentioned as “*Babesia microti*-like piroplasm”, “*Babesia* Spanish dog isolate” and *Babesia* cf. *microti*.

**Electronic supplementary material:**

The online version of this article (10.1186/s13071-019-3385-z) contains supplementary material, which is available to authorized users.

## Background

*Babesia* Starcovici, 1893 and *Theileria* Bettencourt, França & Borges, 1907 are tick-borne protozoan genera classified in the phylum Apicomplexa, class Piroplasmea and order Piroplasmida, which infect domestic and wild animals, and humans, and may cause severe disease. Piroplasmids referred to as *Theileria* (*sensu stricto*) have been originally defined by the presence of a pre-erythrocytic life stage in leukocyte host cells and trans-stadial transmission in ticks. In contrast, schizont propagation is absent in species of *Babesia* (*sensu stricto*) and they display the characteristic of tick transovarial transmission [1, 2]. An additional group of piroplasmids are referred to as *Babesia* (*sensu lato*) since they cannot be assigned to either of the above groups [3].

Molecular phylogeny corroborates the taxonomic entities of *Babesia* (*s.s.*) and *Theileria* (*s.s.*) as each conforms to a monophyletic group referred to as Clade VI and Clade V, respectively [3]. In contrast, *Babesia* (*s.l.*) parasites can be clearly distinguished from the above entities and represent a complex of species that can be assigned to at least two other monophyletic groupings designated Clade I (“*Babesia microti*-like piroplasmids”) and Clade II (Western group) [3]. Domestic dogs and wild canines are infected by several piroplasmid species that can cause severe disease. During the past 30 years, several *Babesia* spp. that infect canines have been described in detail and genetically characterized [4]. As outlined in detail in Baneth et al. [5], equivocal phylogenetic placement has led to a subsequent erroneous taxonomic assignment of this parasite species within the genus *Theileria* as “*Theileria annae* Zahler, Rinder, Schein & Gothe, 2000”. To counter this inaccuracy, the parasite was addressed by a plethora of alternative designations and names such as “*B. microti*-like piroplasm” [6], “*Babesia* Spanish dog isolate” [7], “*Babesia annae*” [8], “*Babesia* (*Theileria*) *annae*” [9] and *Babesia* cf. *microti* [10]. The parasite infects red and gray foxes (*Vulpes vulpes* Linnaeus, 1758 and *Urocyon cinereoargenteus* Schreber, 1775) [9, 11–14], as well as golden jackals (*Canis aureus* Linnaeus, 1758), and domestic dogs (*Canis lupus familiaris* Linnaeus, 1758) [15, 16]; and it is associated with disease in dogs presenting with pale mucous membranes, anemia, anorexia and lethargy [17, 18].

A study published by our group has demonstrated that within the *B. microti*-like piroplasmids (Clade I), a new species named *Babesia vulpes* Baneth, Florin-Christensen, Cardoso & Schnittger, 2015 is positioned in a monophyletic group of *Babesia* parasites that exclusively infect carnivores and is closely related to the monophyletic *B. microti* group. Furthermore, we demonstrated that within the *Babesia-*infecting carnivore group, this parasite can be unequivocally delineated as a distinct species [5]. In the latter study, the name *B. vulpes* had been proposed as a new species designation [5]; nevertheless, as pointed out in a Letter to the Editor of this journal by Harris [19], according to Article 16.4 of the *International Code of Zoological Nomenclature* (ICZN), the naming was not statutory. This is because in order to name a species, the naming publication must contain the fixation of a holotype deposited in a specified collection and a description of the species, preferably containing morphological details. As Harris [19] mentioned, these details, as well as the designation of the proposed name as “sp. nov.” were also missing in the naming of *T. annae* by Zahler et al. [6]; therefore, both these names are currently considered *nomina nuda* (plural for *nomen nudum*, Latin for “naked name”, a name not statutorily in force, but which can be made available in subsequent naming procedures) [19]. According to the glossary of ICZN, a *nomen nudum* is not an available name (in the meaning used in the zoological nomenclature) and therefore the same name may be made available later for the same or a different concept.

The purpose of this study is therefore to further characterize and provide the missing requirements (description, designation of a name-bearing type) in order to establish *B. vulpes* n. sp. as a valid species name.

## Methods

Blood smears fixed with methanol and stained with Hemacolor® (Merck, Darmstadt, Germany) were obtained from the Inno Veterinary Laboratory in Braga, Portugal, and evaluated for parasite morphology by light microscopy. Piroplasm parasites from these smears prepared in 2009 from two Portuguese dogs infected with this parasite then termed *B. microti*-like were previously examined, described and molecularly characterized [17]. The smears were examined by oil immersion microscopy (Zeiss, Jena, Germany) at 1000× magnification. Sizes of parasites were measured using a micrometer. Measurements are in micrometers and are given as the range followed by the mean and standard deviation in parentheses. A stained blood smear from one of these dogs containing the holotype was deposited in the National Natural History Collection of the Hebrew University of Jerusalem, Israel, and the remaining slides containing the paratypes were deposited in the Parasite Collection of the University of Oporto, Portugal.

PCR to amplify the *cox*1 gene was carried out using blood samples from three Israeli red foxes (*V. vulpes*) collected for a hemoparasite survey. The samples had been shown to be infected with the new species by PCR of the *18S* rRNA gene followed by sequencing (GenBank: KJ871347, KJ871348, KJ871349), and for which a nearly complete longer gene sequence (GenBank: KJ871351) derived from one fox had been used in the phylogenetic analysis of Baneth et al. [5]. To this end, a region of the *cox*1 gene was amplified using primers cox1F133 and cox1R11130 essentially as previously described [20]. Conventional PCR was performed in a total volume of 25 μl using the PCR-ready High Specificity mix (Syntezza Bioscience, Jerusalem, Israel) with 400 nM of each primers and sterile DNase/RNase-free water (Sigma, St. Louis, MO, USA). Amplification was carried out using a programmable, conventional thermocycler (Biometra, Göttingen, Germany). PCR products were electrophoresed on 1.5% agarose gels stained with ethidium bromide and evaluated under UV light for the size of amplified fragments by comparison to a 100 bp DNA molecular weight marker. Direct sequencing of PCR allowed determining the *cox*1 nucleotide sequences (GenBank: KX169167, KX169168 and KX169169) and corresponding COX1 amino acid sequences (GenBank: APX55184, APX55185 and APX55186) for subsequent inclusion into phylogenetic analyses.

An alignment of COX1 amino acid sequences of piroplasmid species available on GenBank was done by MUSCLE [21, 22]. Aligned sequences comprised of 26 COX1 sequences including sequences of *B. vulpes* n. sp. that have been derived from three different canine species of distant geographical origin: red fox (*V. vulpes*) from Israel determined for this study as described above, Eurasian golden jackal (*C. aureus*) from Romania (GenBank amino acid sequence ARN62236 corresponding to nucleotide sequence KX712132), and a domestic dog (*Canis lupus familiaris*) from the USA (GenBank amino acid AGF95361 corresponding to nucleotide sequence KC207827). All positions containing gaps and missing data were eliminated, resulting in a final dataset of 293 positions. The JTT + G model with the shape parameter (G = 0.56) was selected based on Akaike information criterion (AIC) and a neighbor-joining tree inferred [23, 24].

## Results

**Family Babesiidae Poche, 1913**

**Genus** ***Babesia*** **Starcovici, 1893**

***Babesia vulpes*** **n. sp.**

***Type-host***: Domestic dog *Canis lupus familiaris* Linnaeus, 1758 (Mammalia: Canidae).

***Other hosts***: Red fox *Vulpes vulpes* (Linnaeus, 1758), gray fox (*Urocyon cinereoargenteus* Schreber, 1775), golden jackal (*Canis aureus* Linnaeus, 1758).

***Type-locality***: City of Braga (41°33′6″N, 8°25′22″W), Portugal.

***Other localities***: Austria [13], Bosnia and Herzegovina [25], Canada [9], Croatia [26], France [27], Germany [28], Great Britain [29], Hungary [10], Israel [14], Italy [30], Romania [16], Slovakia [31], Spain [15, 18], Turkey [32], USA [7, 12].

**Fig. 1:**
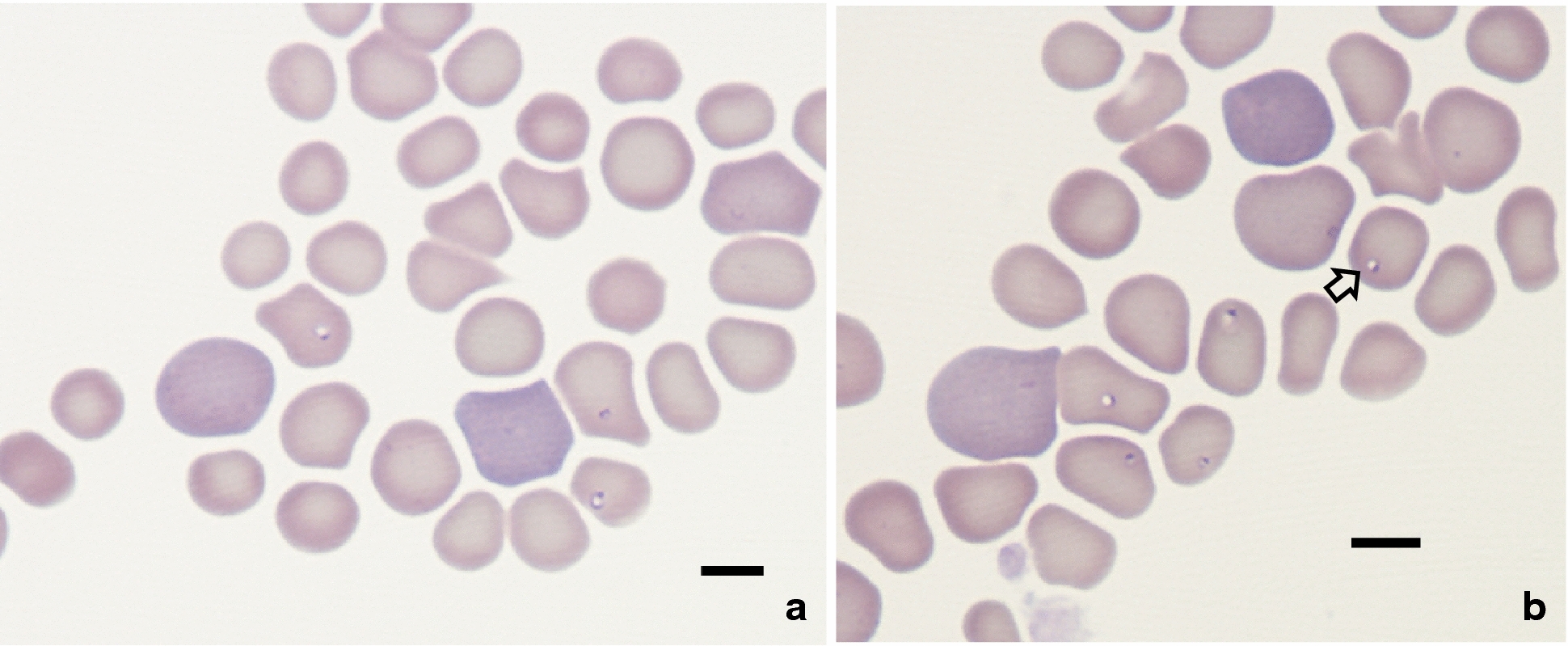
*Babesia vulpes* n. sp., type-material in blood smears from a dog (**a**, **b**). Giemsa staining. The holotype is marked with an arrow in **b**. *Scale-bars*: 5 μm

***Type-material***: A stained thin blood smear from a 4-year-old Portuguese female dog containing the holotype (Fig. 1b) was deposited in the National Natural History Collection of the Hebrew University of Jerusalem, Israel, under the accession number “HUJPROTOZ1001”. Blood smears containing paratypes were deposited at the Parasite Collection, Laboratory of Animal Pathology, CIIMAR-Centro Interdisciplinar de Investigação Marinha e Ambiental (Interdisciplinary Centre of Marine and Environmental Research), University of Oporto, Portugal under the accession number CIIMAR 2016.9. In addition, genomic DNA extracted from the blood of the Portuguese dog and three red foxes infected with the parasite from Israel (foxes nos. 910, 917 and 26217) has been deposited at the Koret School of Veterinary Medicine, Hebrew University of Jerusalem, Rehvot, Israel under the accession numbers 12019–32019.

***Vector***: Unknown. *Ixodes hexagonus* Leach, 1815, *Ixodes ricinus* Linnaeus, 1758, *Ixodes canisuga* Johnston, 1849, *Dermacentor reticulatus* Fabricius, 1794 and *Rhipicephalus sanguineus* Latreille, 1806 are suspected [28, 33–35].

***Representative DNA sequences***: Present study (GenBank: KX169167-KX169169; *cox*1); Baneth et al. [5] and Margalit-Levi et al. [14] (GenBank: KJ871346-KJ871352; *18S* rRNA).

***ZooBank registration***: To comply with the regulations set out in article 8.5 of the amended 2012 version of the *International Code of Zoological Nomenclature* (ICZN) [36], details of the new species have been submitted to ZooBank. The Life Science Identifier (LSID) of the article is urn:lsid:zoobank.org:pub:9A1011D2-063C-4E5A-B74D-DDD89EE0772F. The LSID for the new name *Babesia vulpes* is urn:lsid:zoobank.org:act:DF4C2543-0038-435B-AA52-01A2D9239DB7.

***Etymology***: The species is named after the red fox (*V. vulpes*) considered as the main wildlife host of this parasite. The species name “*vulpes*” is a noun in apposition (ICZN Article 31.1.2).

### Description

***Merozoites*** [Measurements based on 18 parasites; see Fig. 1.] Round to oval-shaped merozoites with an eccentric round nucleus presenting as single or two parasites in erythrocyte. Merozoites measuring 0.8–1.9 (1.33 ± 0.28) in length and 0.7–1.4 (0.98 ± 0.22) in width (*n* = 18), with nuclei measuring 0.4 in diameter (*n* = 4). No tetrad (Maltese cross) shapes observed.

### Differential diagnosis

Intraerythrocytic parasites presented as round to oval-shaped, and an eccentric, basophilic-staining; round nucleus was conspicuous in some parasites (Fig. 1a, b). Of 18 parasites measured, 16 presented as single parasites, whereas the remaining two were located in the same erythrocyte. Parasites occupied only a small portion of the erythrocyte and were reminiscent of the pyriform and ring shapes described for other small-form *Babesia* species that infect dogs [37, 38]; however, no tetrad (Maltese cross) shapes were seen.

The morphological shape of *B. vulpes* n. sp. described here from dog erythrocytes is similar to the ring and pyriform shapes described for other small, canine-infecting *Babesia* spp. [38–40]. Nevertheless, the merozoites of *B. vulpes* n. sp. measuring on average 1.33 × 0.98 µm, are distinctly smaller than the merozoites of *Babesia vogeli* Reichenow, 1937, *Babesia canis* Pianna & Galli-Vallerio, 1895 and *Babesia rossi* (Nuttal, 1910) Wenyon, 1926, with size typically within the range of 4.5–5.0 × 2.0–2.5 µm (as summarized in [4]). They are also smaller than the ring forms described for *Babesia conradae* Kjemtrup, Wainwright, Miller, Penzhorn & Carreno, 2006, which measure 2.2 × 1.85 µm, and are closer in size to the pyriform shapes of *B. conradae* which measure 1.38 × 0.66 µm [38]. However, in contrast to *B. conradae*, no tetrad (Maltese cross) forms were observed in *B. vulpes* n. sp. *Babesia gibsoni* Patton, 1910, another small-form *Babesia* of dogs, which is also not reported to produce tetrads, is described as being considerably larger than *B. vulpes* n. sp. with the ring shape measuring 2.71 × 1.61 µm and the pyriform shape measuring 2.1 × 0.94 µm [40], or according to a different report, 1.9 × 1.2 µm, without a distinction between the shapes [39]. The above comparisons indicate that *B. vulpes* n. sp. is a distinct form consistent with the small-form piroplasms of canines. However, *B. vulpes* n. sp. tends to be smaller than *B. conradae* and *B. gibsoni* and has not been reported to form tetrads, thus further distinguishing it from *B. conradae*.

We consider that previous reports with morphological details on intraerythrocytic piroplasm forms seen by light microscopy in stained blood smears of synonyms of *B. vulpes* n. sp., e.g. “*T. annae*” [6, 18, 41, 42], “*B. microti*-like piroplasm” [6, 15, 17, 18, 43–45], and “*Babesia* (*Theileria*) *annae*” [9], from the domestic dog [6, 15, 17, 18, 43, 44] and the red fox [9, 45], actually represent *B. vulpes* n. sp. These reports describe intraerythrocytic ring-shaped or oval to round organisms morphologically compatible with small piroplasms [9, 18, 42, 45] which are 1–2 µm in diameter [6, 9, 42], as found for *B. vulpes* n. sp., and having a dark-staining dot-shaped nucleus [42]. The small piroplasms reported were present mostly as single parasites in erythrocytes and rarely as two intracellular organisms, and located centrally to paracentrally in their host erythrocytes [6, 9, 15]. PCR and sequencing of the parasites seen by microscopy in all of these reports indicated they have identical sequences to *B. vulpes* n. sp. and its synonyms [6, 9, 15, 17, 18, 41–45].

### Molecular phylogeny

**Fig. 2:**
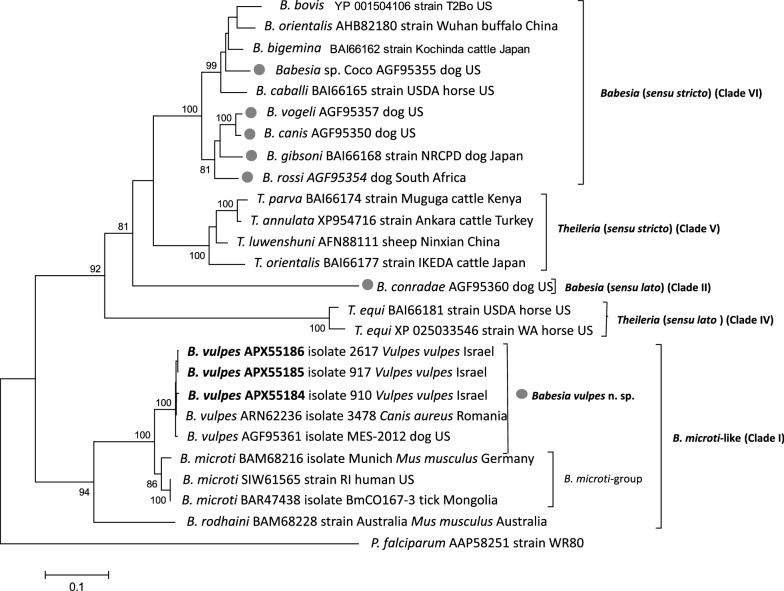
Neighbor-joining tree of COX1 amino acid sequences of *Babesia vulpes* n. sp. and other piroplasmid species. Sequences analyzed in the context of this study are designated by bold accession numbers of taxon labels. Clade designations are presented as defined previously [3, 50]. The percentage of replicate trees as determined by 1000 replicates of a bootstrap test are shown next to the branches. A *Plasmodium falciparum* COX1 sequence has been included as the outgroup. The scale-bar represents the evolutionary distance in the units of the number of amino acid substitutions per site. Gray dots designate *Babesia* species that infect domestic dogs [51]

Phylogenetic analysis of amino acid COX1 sequences for *Theileria* spp. and *Babesia* spp. resulted in a tree that recovers Clades I [*Babesia* (*s.l.*), *Babesia microti*-like group], II [*Babesia* (*s.l.*) of the Western Clade], IV [*Theileria equi* (Laveran, 1901) Melhorn & Schein, 1998], V [*Theileria* (*s.s.*)], and VI [*Babesia* (*s.s.*)] as previously reported by Schreeg et al. [46] and based on *18S* rRNA gene sequences by Schnittger et al. [3] (Fig. 2). Canine-infecting *Babesia* sp. Coco, *B. vogeli*, *B. rossi*, *B. canis* and *B. gibsoni* were clustered with strong support into the *Babesia* (*s.s.*) Clade VI (bootstrap support, bs = 100), whereas canine-infecting *B. conradae*, segregated into the well-supported Clade II [*Babesia* (*s.l.*) of the Western Clade, bs = 81]. Importantly, the strongly supported joint placement of COX1 sequences of isolates from geographically distant locations and diverse canine hosts (*V. vulpes* from Israel, *C. aureus* from Romania and *C. l. familiaris* from the USA) attests the distinct species status of *B. vulpes* n. sp. (bs = 100). The clade to which *B. vulpes* n. sp. is most closely related, yet can be clearly distinguished from, is the strongly supported *B. microti* group (bs = 86). Furthermore, *Babesia rodhaini* Van den Berghe, Vincke, Chardome & Van den Bulcke, 1950 represented a strongly supported sister species to the clade comprising of *B. vulpes* n. sp. and the *B. microti* group. The results based on the COX1 amino acid sequences coincide and support the previously presented results on the species identity of *B. vulpes* n. sp. by phylogenetic analysis of *18S* RNA and *β-tubulin* gene sequences [5]. In addition, a neighbor-joining tree based on 25 *cox*1 nucleotide sequences with a final dataset of 879 positions of *B. vulpes* n. sp. and other piroplasmid species was inferred and corroborated results obtained by COX1 amino acid sequences. Specifically, the same topology and an identical bootstrap support were determined for the corresponding relevant clades of the trees inferred by amino acid and nucleotide sequences [*B. microti* group/*B. vulpes* n. sp. clade (bs = 100) and (*B. vulpes* n. sp. clade (bs = 100)] (Additional file 1: Figure S1).

## Discussion

This study establishes *B. vulpes* n. sp. as a new taxon fulfilling the requirements of the ICZN guidelines. A morphological description with measurements of the parasite forms in canine erythrocytes and the deposition of the holotype and paratypes in suitable collections have been made in compliance with the ICZN guidelines [36]. The generic placement of *B. vulpes* n. sp. is derived from the molecular phylogenetic analysis of the *18S* RNA and *β-tubulin* genes, and COX1 protein sequences, whereas the species name has been chosen because the red fox (*V. vulpes*) is considered the main natural host of this piroplasmid (see also [5]). As mentioned above, according to the ICZN regulations, “*T. annae*” [6] is considered a non-available name (*nomen nudum*), which has never been valid in terms of the Code, and thus the principle of priority does not apply in this case. Accordingly, as previously stated [19], the species name “*annae*” does not need to be carried into the proposed species designation. The renaming of “*T. annae*” as *B. vulpes* n. sp. should now replace the use of all synonyms for this species, such as “*B. microti*-like piroplasm”, *Babesia* cf. *microti*, “*B. annae*” and “*Babesia* Spanish dog isolate”, thus ending the confusion when referring to this parasite species. Furthermore, in accordance with recent findings on the molecular phylogeny of this and other piroplasmid species, the proposed name clearly distinguishes this parasite from species of the genus *Theileria* Bettencourt, França & Borges, 1907.

The COX1 has been increasingly applied in molecular phylogenetic studies of piroplasmids [20, 46, 47]. The phylogenetic analysis using COX1 demonstrated that *B. vulpes* n. sp. does not segregate into *Theileria* (*s.s.*) (Clade V) nor into *Babesia* (*s.s.*) (Clade VI), but into a group of *Babesia* (*s.l.*) species that is placed into Clade I (*B. microti*-like parasites or Archaeopiroplasmida; see [11]). Within Clade I, *B. vulpes* n. sp. is strongly supported as a distinct species of a subclade of *Babesia* (*s.l.*) species that has so far been found to exclusively infect carnivores of the families Mustelidae and Canidae. The subclade including *B. vulpes* n. sp. can be clearly distinguished from the subclades of the *B. microti* group and *B. rodhaini* together forming Clade I of *B. microti*-like piroplasmids (Fig. 2; [3]). As previously outlined in detail, the *18S* rRNA and *β-tubulin* gene phylogenetic analyzes are in accordance with this result [5]. *Babesia vulpes* n. sp. is the first species defined within its own subclade group and it is expected that additional species in this group will be described in the future (see also [5]).

Overall, the congruent phylogenetic analysis of the *18S* and *β-tubulin* genes and the COXI protein-sequence encoded by the mitochondrial genome, and the fact that *B. vulpes* n. sp. has not been shown to infect rodents and humans, distinguishes it as a species from the zoonotic *B. microti* located in the *B. microti* group (Fig. 2). Furthermore, *B. microti* from mice belonging to the zoonotic *B. microti* group was not found to be infectious to dogs, pigs, chicken and goats in an experimental transmission study, while it was infectious to rats [48].

The mode of transmission and tick vectors of *B. vulpes* n. sp. have not been determined yet. Although the DNA of this parasite has been detected in several tick species (reviewed in [5]), including *I. hexagonus*, which was proposed as a vector [49], and *D. reticulatus* [35], no study to date has provided sufficient proof for the vectorial capacity of any particular tick species, and further research is needed to elucidate this issue.

## Conclusions

The fixation of holotype and the morphological description and differentiation of the new species provided here establish the species name *B. vulpes* n. sp. by fulfilling the ICZN requirements for description of a new species. The name *B. vulpes* n. sp. should replace all the synonyms that have been used for this parasite including “*Theileria annae*”, “*Babesia annae*”, “*B. microti*-like piroplasm”, *Babesia* cf. *microti* and “*Babesia* Spanish dog isolate”.

## Additional file

**Additional file 1: Figure S1.** A neighbor-joining tree of 25 *cox*1 nucleotide sequences of *B. vulpes* n. sp. and other piroplasmid species. Clade designations are presented as defined previously [3, 50]. After alignment of nucleotide sequences, all positions containing gaps and missing data were eliminated, resulting in a final dataset of 879 positions. The T92 + G model with the shape parameter (G = 0.42) was selected based on Akaike information criterion (AIC) and the neighbor-joining tree inferred [23, 24]. The percentages of replicate trees as determined by 1000 replicates of a bootstrap test are shown next to the branches. A *Plasmodium falciparum cox*1 sequence has been included as the outgroup. The scale-bar represents the evolutionary distance in the units of the number of nucleotide substitutions per site. Gray dots designate *Babesia* spp. that infect domestic dogs [51].

## Abbreviations

COX1: cytochrome *c* oxidase 1
ICZN: *International Code of Zoological Nomenclature*
PCR: polymerase chain reaction
