# Supplementary material for: Establishment of Babesia vulpes n. sp. (Apicomplexa: Babesiidae), a piroplasmid species pathogenic for domestic dogs
Source: Parasit Vectors. 2019 Mar 26;12:129. doi: 10.1186/s13071-019-3385-z (PMC6434798; doi:10.1186/s13071-019-3385-z)
Supplement: Supplementary file 1 — Additional file 1: Figure S1. A neighbor-joining tree of 25 cox1 nucleotide sequences of B. vulpes n. sp. and other piroplasmid species. Clade designations are presented as defined previously [3, 50]. After alignment of nucleotide sequences, all positions containing gaps and missing data were eliminated, resulting in a final dataset of 879 positions. The T92 + G model with the shape parameter (G = 0.42) was selected based on Akaike information criterion (AIC) and the neighbor-joining tree inferred [23, 24]. The percentages of replicate trees as determined by 1000 replicates of a bootstrap test are shown next to the branches. A Plasmodium falciparum cox1 sequence has been included as the outgroup. The scale-bar represents the evolutionary distance in the units of the number of nucleotide substitutions per site. Gray dots designate Babesia spp. that infect domestic dogs [51]. [file 13071_2019_3385_MOESM1_ESM.pptx]

## Slide 1
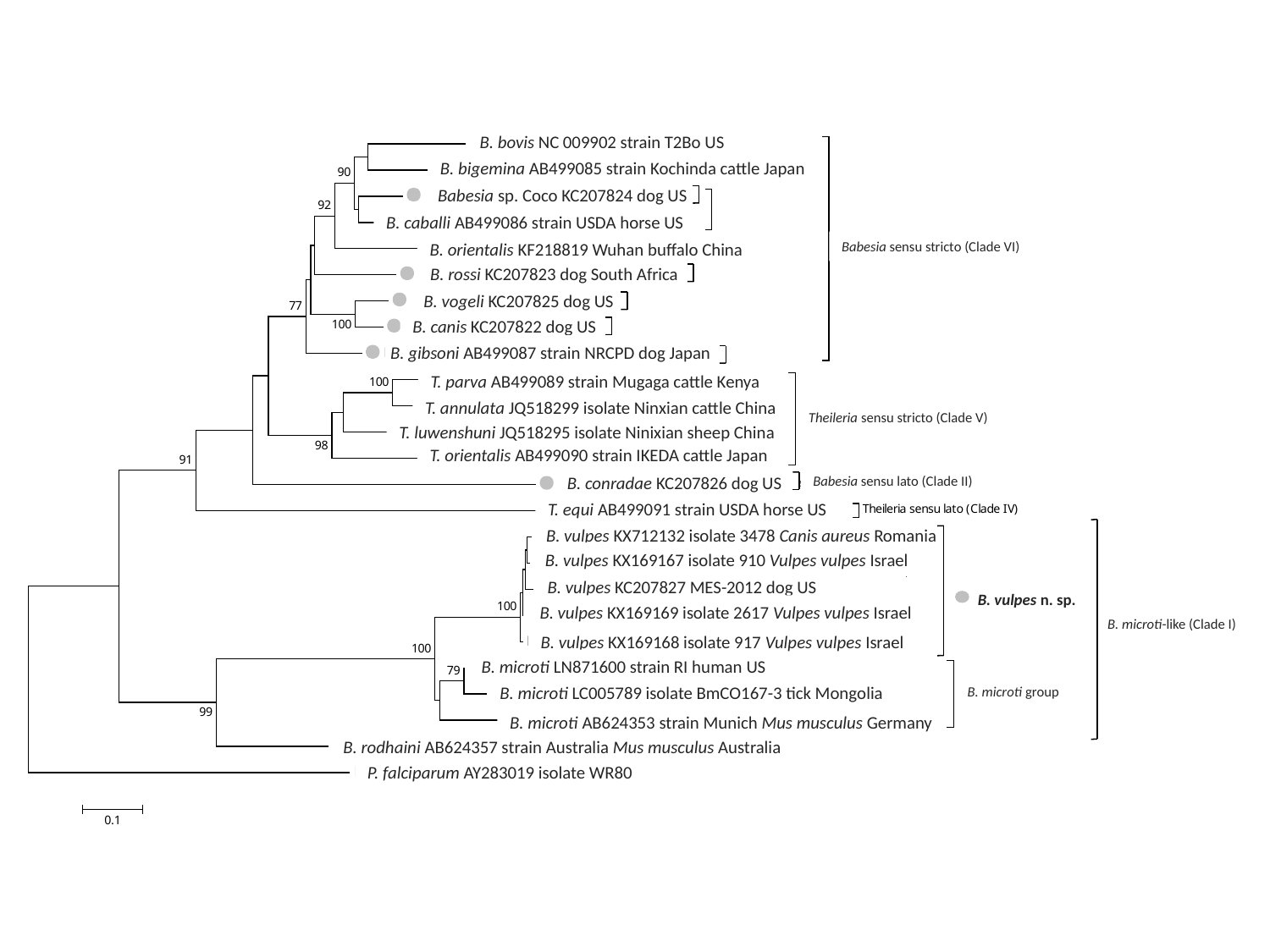

B. bovis NC 009902 strain T2Bo US
B. bigemina AB499085 strain Kochinda cattle Japan
Babesia sp. Coco KC207824 dog US
B. caballi AB499086 strain USDA horse US
Babesia sensu stricto (Clade VI)
B. orientalis KF218819 Wuhan buffalo China
B. rossi KC207823 dog South Africa
B. vogeli KC207825 dog US
B. canis KC207822 dog US
B. gibsoni AB499087 strain NRCPD dog Japan
T. parva AB499089 strain Mugaga cattle Kenya
T. annulata JQ518299 isolate Ninxian cattle China
Theileria sensu stricto (Clade V)
T. luwenshuni JQ518295 isolate Ninixian sheep China
T. orientalis AB499090 strain IKEDA cattle Japan
B. conradae KC207826 dog US
Babesia sensu lato (Clade II)
T. equi AB499091 strain USDA horse US
B. vulpes KX712132 isolate 3478 Canis aureus Romania
B. vulpes KX169167 isolate 910 Vulpes vulpes Israel
B. vulpes KC207827 MES-2012 dog US
B. vulpes n. sp.
B. vulpes KX169169 isolate 2617 Vulpes vulpes Israel
B. microti-like (Clade I)
B. vulpes KX169168 isolate 917 Vulpes vulpes Israel
B. microti LN871600 strain RI human US
B. microti LC005789 isolate BmCO167-3 tick Mongolia
B. microti group
B. microti AB624353 strain Munich Mus musculus Germany
B. rodhaini AB624357 strain Australia Mus musculus Australia
P. falciparum AY283019 isolate WR80
